# Integrating Domestic Violence Education into the Pharmacy Curriculum

**DOI:** 10.3390/pharmacy13010008

**Published:** 2025-01-22

**Authors:** Harjit K. Khera, Rita Wardan, Hiu Tek Wu, Andy Ling, Suzanne M. Caliph

**Affiliations:** Faculty of Pharmacy and Pharmaceutical Sciences, Monash University, Parkville, VIC 3052, Australia; harjit.khera@monash.edu (H.K.K.); rita.wardan@monash.edu (R.W.); hwuu0065@student.monash.edu (H.T.W.); alin0033@student.monash.edu (A.L.)

**Keywords:** domestic violence, pharmacy education, healthcare, curriculum integration, pharmacists, public health, training, interdisciplinary collaboration

## Abstract

Domestic violence (DV) is a pervasive issue with significant implications for public health, yet it remains under-addressed in healthcare systems. Pharmacists, as accessible healthcare providers, are in a unique position to identify and support individuals affected by DV, but training in this area is often lacking in pharmacy education. This study explores the challenges of and opportunities for integrating DV education into the pharmacy curriculum by interviewing twelve pharmacy educators from Monash University. Using semi-structured interviews, data were analyzed thematically to identify key barriers and facilitators. The findings highlight several benefits of integrating DV education, including pharmacists’ accessibility to patients and their ability to play a vital role in recognizing and responding to DV. However, challenges such as time constraints, lack of confidence, insufficient training, and perceived limitations on pharmacists’ scope of practice were noted. Ethical and legal concerns regarding pharmacists’ roles in DV cases were also identified. This study concludes that integrating DV education into pharmacy curricula is essential; however, it requires significant barriers to be overcome, including the need for specialised training and collaboration with DV experts. This study recommends interdisciplinary workshops and greater faculty support to equip future pharmacists with the necessary skills to address DV effectively.

## 1. Introduction

Domestic violence (DV) remains a pervasive societal issue with profound implications for public health and individual well-being. The World Health Organization (WHO) defines domestic violence as “the intentional use of physical force, threats, intimidation, or psychological abuse by a family member, intimate partner, or caregiver to control another person’s behaviour”. It encompasses various forms of abuse, including physical, sexual, emotional, and economic, and affects individuals across all demographics, regardless of age, gender, race, or socioeconomic status [1]. One in three women are anticipated to experience physical or sexual violence in their lifetime, often at the hands of an intimate partner, reflecting the widespread nature of domestic violence and its significant impact on women’s health and well-being globally. The impact of domestic violence (DV) on both physical and mental health outcomes has been exacerbated by the rise in DV cases during the COVID-19 pandemic, increasing vulnerabilities across multiple demographics.

From 2021 to 2022, in Australia, an estimated one in four women and one in eight men experienced violence by an intimate partner or family member [2]. DV-affected individuals are significantly more likely to suffer from long-term mental health problems, including depression, anxiety, and post-traumatic stress disorder (PTSD), and are less likely to adhere to treatment regimens, further complicating healthcare management [3,4,5]. The use of pain medicines (including opioids) and psychotropic medicines (e.g., antidepressants, sedatives) are also reported to be highly prevalent in this population [6,7,8]. Despite its prevalence and profound impact on health outcomes, and the rise in DV cases as a consequence of the pandemic, DV remains underreported and inadequately addressed within healthcare systems, often due to the societal stigma, lack of awareness, and a general underutilisation of available resources [9,10,11].

Pharmacists, as readily accessible healthcare professionals with expertise in medication management and patient care, are in a unique position to identify and assist individuals or families affected by DV [4]. DV-affected individuals often do not seek help, and when they do, it is usually when they are presented with serious physical injuries in hospitals [12]. Community pharmacists are frequently an underutilised resource in the management of domestic violence. They have the potential to play a significant role in identifying signs of DV, offering support, and connecting patients with appropriate resources and services.

Pharmacy professionals worldwide have recognised the need to address DV more proactively within healthcare systems, yet there remains a gap in the formal training on the topic. In other healthcare professions, such as nursing and social work, there have been initiatives to integrate DV education into curricula, however, pharmacy has lagged behind in this area [6]. Understanding how DV education has been incorporated into other healthcare training can provide valuable insights.

The requirements for reporting suspected cases of DV vary across countries, with some having established policies and others enforcing legal obligations. In Australia, for example, mandatory reporting laws are in place across all states and territories to address child abuse, including situations where children are exposed to DV. However, when it comes to DV involving adults, the reporting requirements are more complex and differ between states and territories [2]. Best practices involve encouraging adult victims to seek support such as counselling, legal advice, or police assistance and calling emergency services if there is an immediate threat to safety.

Given the vital role of pharmacists in healthcare and the significant impact of DV on patient health, it is imperative that pharmacy students receive appropriate education and training to recognise and assist DV-affected patients in their practice. However, integrating DV education into the pharmacy curriculum may present challenges including limited time and competing content priorities, and the need for academic capacity, such as faculty expertise in this area.

This study aims to identify key challenges and opportunities in integrating DV education into the pharmacy curriculum by exploring the perspectives of pharmacy educators who are practising pharmacists. Furthermore, it seeks to explore the perceptions and attitudes of pharmacy educators regarding the relevance and importance of DV education in pharmacy practice. By identifying gaps and opportunities for integrating DV training, we hope to contribute to the preparation of future pharmacists who are equipped to address DV effectively and compassionately within their professional practice of patient safety and quality care.

## 2. Materials and Methods

### 2.1. Ethics Approval

Ethics approval for this study was received from the Monash University ethics committee (2024-40480-10252).

### 2.2. Participants and Recruitment

The participants of this study included 12 pharmacy practitioner educators from Monash University’s Faculty of Pharmacy and Pharmaceutical Sciences (FPPS). The recruitment process used purposive sampling, with personalised email invitations sent to participants that included a comprehensive explanatory statement outlining this study’s purpose, procedures, risks, and benefits, and ethical consideration of this study to ensure that participants had all the information needed to make an informed decision about whether to participate.

### 2.3. Interviews

Once participants expressed their interest and availability, interviews with each participant were scheduled and conducted virtually via the Zoom video-conferencing platform between February and March 2024.

Prior to commencing each interview, verbal consent was explicitly obtained from the participants. During the interviews, both audio and video recordings were captured to ensure comprehensive data capture. These recordings were subsequently transcribed verbatim and subjected to thematic analysis.

A semi-structured interview guide was developed in alignment with this study’s aims and objectives, comprising six key thematic areas: General Understanding, Challenges, Opportunities, Curricular Impact, Assessment and Evaluation, as well as Resources and Support.

### 2.4. Data Analysis

The digitally recorded interviews were transcribed verbatim. NVivo 11 (QSR International 2020) was used for data management and thematic analysis. Initially, two researchers independently analysed the first four interview transcripts using an inductive coding process [13,14] to generate a comprehensive list of codes. Any discrepancies in coding were carefully reviewed, discussed, and resolved, leading to the development of a hybrid model combining inductive and codebook thematic analysis. In subsequent rounds, the researchers revisited and re-coded the initial interviews based on this updated framework, actively seeking new insights. The individual coding files were then integrated to create a cohesive final coding scheme, from which key themes emerged. The analysis reached inductive thematic saturation when no new codes appeared in later reviews. To ensure reliability, intercoder agreement was assessed using Cohen’s kappa, with a target threshold of 0.70 to indicate substantial agreement [15]. Collaborative discussions between the researchers to reach consensus helped to refine the coding scheme and improve consistency. The coding process was streamlined by eliminating redundant or infrequently used codes and merging similar ones. Once intercoder reliability met the established threshold, subsequent interviews were coded by a single investigator, adhering to the finalised coding scheme. This iterative process ensured the robustness and integrity of the data analysis framework.

## 3. Results

Twelve interviews with pharmacy educators were conducted for the study with an average duration of 20 min. Participants included 12 pharmacy educators who were also qualified pharmacists. The participants held diverse roles as pharmacy educators, ranging from teaching associates to lecturers and course directors, with educators’ years of experience spanning from 3.5 years to 20 years.

The analysis of the interview transcripts revealed five key themes: Barriers to integrating DV into the curriculum, Benefits of integrating DV into the curriculum, Ethical and legal considerations related to a Pharmacist’s role and responsibilities, the understanding of DV related to a Pharmacist’s role and responsibilities, and Pharmacist resources on DV (Table 1). Subcategories are italicised in the following text, with representative quotations for each subcategory listed in Appendix A. Each educator is also assigned a reference number for clarity.

### 3.1. Benefits of Integrating DV into the Curriculum

The first major theme that emerged was the Benefits of Integrating DV into the pharmacy curriculum. One of the key benefits of integrating domestic violence (DV) education into the pharmacy curriculum, as highlighted by the interviewees, is the accessibility of pharmacists to the community. Pharmacists are often the most accessible healthcare practitioners, allowing patients to seek assistance without the need for an appointment. They may be trained to recognise the signs of domestic violence and identify issues that a patient may be facing, including its impact on medicine use and misuse. Interviewees noted that this accessibility empowers pharmacists to expand their roles and to know whom to refer patients to for further support.

Regarding recommendations for curriculum integration, participants suggested implementing interdisciplinary workshops that involve students from various healthcare professions, such as nursing medicine and social work. This collaborative approach would help students to learn how different disciplines can support a patient and understand the legalities involved in addressing DV—specifically, what actions they can and cannot take. Additionally, educating students about the warning signs of domestic violence and the appropriate steps to take when those signs are observed was emphasised as essential training.

With regard to recommendations for curriculum integration, interviewees highlighted that the scope of pharmacy practice continues to evolve with opportunities for pharmacists to collaborate with and support other healthcare professionals specialising in DV to provide comprehensive care to affected patients. For example, mental health pharmacists may play a critical role by collaborating with outreach teams to support patients dealing with domestic violence and mental health issues. Patients with mental health or chronic pain conditions often have unique pharmaceutical needs, such as atypical doses of medicines or staged supplies, which requires pharmacists to adjust their counselling on medicine storage and access, especially in households with potential abuse risks.

### 3.2. Barriers to Integrating DV into the Curriculum

The second prominent theme identified was Barriers to Integrating DV into the Curriculum. Conversations in this area addressed several challenges, including pharmacists’ lack of confidence or experience, and the perception that addressing domestic violence falls outside the pharmacist’s current scope of practice. Additionally, time constraints for including extra materials in the pharmacy curriculum were frequently noted.

The lack of confidence and experience emerged as a recurring concern among eight interviewees. Many expressed apprehension about teaching students how to recognise and intervene in cases of domestic violence, as well as how to respond appropriately once signs are identified. Concerns were raised about the potential stress that DV education could place on students, particularly those who may have experienced domestic violence themselves. Additionally, interviewees indicated that pharmacy educators might not be well-equipped and suggested that DV-specialised professionals would be needed to effectively teach this content.

Many participants expressed concerns about their lack of confidence in teaching this subject, often questioning whether it is more appropriately addressed by social workers, police, or other healthcare professionals. They also noted that the educational resources currently available on DV are not tailored to pharmacists. 

A recurring sub-theme in the interviews was the challenge of managing time constraints within the already-rigorous pharmacy curriculum. Integrating domestic violence (DV) education would likely require reducing or the removal of certain topics from the curriculum, depending on the emphasis placed on DV. Additionally, there are significant considerations regarding the time and resources needed to develop a high-quality module or content on this subject. Developing a new curriculum that meets educational standards is a substantial undertaking, and educators are often pressed for time as it is.

### 3.3. Ethical or Legal Consideration Related to a Pharmacists’ Role and Responsibilities

The next theme, Ethical and Legal Considerations, encompassed various sub-themes related to the ethical responsibilities that pharmacists must navigate, as well as relevant legal considerations. Some participants also expressed concerns about a lack of clarity regarding these ethical and legal considerations, which may impact their ability to engage effectively with patients experiencing DV issues.

One of the key ethical responsibilities highlighted by the interviewees is the need for pharmacists to possess the appropriate skills and knowledge to recognise signs of domestic violence and to make referrals when necessary. They emphasised the importance of being aware of evidence-based guidelines and stressed that students should be educated about these resources to inform their practice and decision-making.

Regarding legal considerations, the interviewees noted that their expertise lies in pharmacy, rather than law. From a legal perspective, it is crucial not to overstep these boundaries. Interviewees highlighted that pharmacists would have legal reporting obligations related to domestic violence (DV). In addition, interviewees expressed uncertainty regarding the clarity of ethical and legal considerations that pharmacists must navigate when addressing domestic violence (DV). They expressed concerns about potential legal challenges that could arise from advising students on how to handle DV cases, fearing that it could lead to complications. Instead, they recommended that students should be trained to recognise, refer, and seek guidance from professionals specifically trained to support DV-affected individuals.

### 3.4. Understanding of DV Related to a Pharmacists’ Role and Responsibilities

The fourth theme, Understanding of Domestic Violence in relation to a pharmacist’s role and responsibilities, encompassed discussions on the definitions and interpretations of DV. Participants shared their perspectives, highlighting the complexities involved in understanding this critical issue. Each interviewee offered their own definitions and interpretations of DV. Overall, it was noted that DV could manifest as verbal, psychological or physical abuse, typically occurring within the home and often involving a family member as the perpetrator.

### 3.5. Pharmacist Resources on DV

The final theme, Pharmacist Resources on DV, focused on interviewees’ familiarity with existing resources related to domestic violence. Some participants demonstrated a strong understanding of available resources, while others expressed uncertainty, highlighting the need to enhance awareness and accessibility of support materials.

The interviewees discussed their familiarity with available resources on domestic violence (DV), including a hotline for clients, various training resources, and government-provided guidelines aimed at educating healthcare professionals about DV. Some also suggested that conducting a Google search could help to identify additional references to support patients effectively. Some participants expressed uncertainty about the resources available for DV, noting that it was outside their area of expertise. However, they acknowledged the existence of numerous resources.

## 4. Discussion

This study examined the challenges, opportunities, and attitudes related to integrating domestic violence education into the pharmacy curriculum, uncovering several key themes that highlighted the complexities of addressing this critical issue in pharmacy education. Our findings highlight the evolving role of pharmacists as accessible healthcare providers uniquely positioned to identify and respond to domestic violence, yet they also reflect significant challenges in equipping educators and students to take on this responsibility.

The interviews revealed five key themes: Benefits of Integrating DV into the Curriculum, Barriers to Integrating DV into the Curriculum, Ethical and Legal Considerations Related to a Pharmacist’s Role and Responsibilities, Understanding of DV Related to a Pharmacist’s Role and Responsibilities, and Pharmacist Resources on DV. Participants highlighted that pharmacists were often the first point of contact as healthcare professionals in the community readily and freely accessible to patients and could play a critical role in identifying and referring individuals experiencing DV [4]. The accessibility of pharmacists was noted as a major benefit of integrating DV education into pharmacy practice, aligning with the growing recognition of the role of pharmacists in public health [4].

However, barriers such as a lack of confidence, insufficient training, and perceived limitations on pharmacists’ scope of practice emerged as significant challenges [16]. Educators expressed concerns about their preparedness to teach DV-related content, emphasising the need for collaboration with professionals trained in DV to strengthen the curriculum [17].

The integration of DV education into the pharmacy curriculum is vital, as it addresses a critical gap in healthcare education [18]. Our findings suggest that incorporating interdisciplinary workshops, as proposed by study participants, could enhance students’ understanding of how various healthcare professionals could work together to support victims of DV [19,20]. This approach not only fosters a comprehensive understanding of patient-centred care, but also prepares pharmacy students to engage more effectively with vulnerable populations.

Moreover, addressing the ethical and legal responsibilities associated with handling DV cases is essential [21]. As highlighted in the results, pharmacists need to be equipped with the knowledge and skills to recognise signs of DV and understand their ethical and legal obligations. This necessitates a curriculum that combines theoretical knowledge with practical guidance, enabling students to navigate these complex situations safely and effectively.

Research on DV education for pharmacy professionals is limited; however, existing studies suggest that pharmacy students often feel inadequately prepared to address DV in practice and lack the confidence to intervene [6,22]. Furthermore, evidence emphasises the importance of embedding DV education early in pharmacy programs, as this timing fosters the development of positive attitudes toward screening and intervention. Integrating partner violence education into pharmacy curricula not only benefits students’ knowledge and confidence, but also enhances the quality of care provided to patients experiencing DV. A stronger focus on DV training during pharmacy education may improve students’ ability to identify at-risk individuals and act appropriately within their professional role.

While this study provides insights into pharmacy educators’ perception of DV education, future research should explore the perspectives and experiences of pharmacy students and pharmacists in practice settings. Understanding their views and experiences can provide deeper insights into the barriers and facilitators of DV education in practice. Furthermore, longitudinal studies examining the impact of DV education on pharmacy practice and patient outcomes would be beneficial in assessing the effectiveness of curriculum changes.

Additionally, exploring partnerships with organisations that specialise in DV support could enhance training resources and provide valuable real-world contexts for students. Incorporating feedback from these organisations may lead to a more robust and responsive curriculum that reflects the complexities of DV issues.

Furthermore, the need for pharmacists to undertake mental health training is crucial. Domestic violence and mental health are deeply interconnected public health issues. Compared with the general population, individuals with a history of domestic violence experienced higher rates of anxiety, depression, and post-traumatic stress disorder (PTSD) [23,24,25]. Mental health conditions often coexist with domestic violence, and pharmacists are in a unique position to identify and support individuals facing both. Including mental health training as part of the pharmacy curriculum can better equip students to address these interconnected issues. The training should aim to enhance pharmacists’ skills in recognizing signs of mental health concerns, communicating effectively with patients, and facilitating appropriate referrals to mental health professionals. Such an approach would strengthen the overall capacity of pharmacists to support victims of domestic violence while ensuring comprehensive care for patients with mental health needs. Consistent with this suggestion, a study by Crespo-Gonzalez et al. [26] highlighted the importance of mental health training in enhancing the knowledge, skills, and confidence of pharmacy professionals to deliver mental health care in community settings. In another study [27], the integration of Mental Health First aid training into the pharmacy curriculum was shown to be associated with significant increases in empathy and self-efficacy of student pharmacists to support individuals with mental health crises. It was also suggested that training healthcare professionals on intimate partner violence could enhance practitioner’s knowledge, attitudes, and perceived readiness to respond to those affected compared with no training [28]. Ethical considerations remained paramount throughout the study, with stringent adherence to protocols ensuring participant confidentiality and anonymity. This study has several limitations, including the limited sample size and the focus on a single institution, both of which may restrict the generalisability of the findings. Additionally, the sample’s homogeneity could impact the applicability of the results to other pharmacy education contexts and programs. The virtual interview format may have influenced the depth and richness of the data collected due to variations in interview dynamics. These factors were addressed with sensitivity and transparency in the interpretation of results, highlighting the need for caution when extrapolating the findings to broader populations. Future studies should aim to include a more diverse participant pool and consider alternative interview formats to enhance the robustness of the data collected.

## 5. Conclusions

This study highlights the importance of integrating domestic violence (DV) education into the pharmacy curriculum, as pharmacists are in a unique position to identify and assist victims. The findings highlight barriers such as insufficient training and a lack of confidence, which need to be addressed to ensure effective integration. Additionally, incorporating mental health training is crucial, given the overlap between DV and mental health issues. Interdisciplinary collaboration and partnerships with DV support organizations can further enhance training. Future research should explore the perspectives of pharmacy students and practitioners, as well as assess the long-term impacts of DV education on practice and patient outcomes. By addressing these challenges, pharmacy education can better prepare pharmacists to support vulnerable populations.

## Figures and Tables

**Table 1:** Themes and subthemes.

Main Theme	Subcategory (Number of Participants Per Sub-Theme)
Benefits of integrating DV into the curriculum	Accessibility of pharmacists to the community (10) Recommendations for curriculum integration (10) Relevance of pharmacist’s role (11)
Barriers to integrating DV into the curriculum	Lack of confidence or experience (8) Outside of pharmacists’ current scope of practice (11) Time constraints (11)
Ethical or legal consideration related to a Pharmacist’s role and responsibilities	Ethical responsibilities (8) Legal considerations (9) Lack of clarity on ethical and legal considerations (3)
Understanding of DV related to a Pharmacist’s role and responsibilities	Definitions and interpretations of DV (12)
Pharmacist resources on DV	Familiarity with available resources (8) Uncertainty about available resources (4)

## Data Availability

The original contributions presented in this study are included in the article. Further inquiries can be directed to the corresponding author.

## Appendix A

Themes, subthemes, and representative quotes identified from interviews

**Main Theme**	**Subcategory**	**Exemplar Quote**
Benefits of integrating DV into the curriculum	Accessibility of pharmacists to the community (10)	“And I think that’s important. And it’s one of the range of potential interventions that pharmacists can make, in terms of if they are trained to recognize signs of domestic violence, for example. Also considering that particularly in community pharmacy, you do develop relationships and rapport with healthcare consumers or patients over time. And so, that can be advantageous in terms of picking up any changes in behavior that may be indicative of domestic violence issues. So I see it being very beneficial, I guess particularly in the community setting, but possibly in the hospital setting as well, considering that victims of domestic violence may very well end up in hospital.” [educator 02] “especially, you know, within community pharmacy, where you can be the primary point of contact with some people. I think it’s important in case, you know, you have patients or people that present to the pharmacy that require support. Especially considering pharmacy as well can be very accessible to people (for) a person that’s experiencing domestic violence—it might be an easy point of contact. For them to just sort of slot into, rather than perhaps, if they were to make an appointment with a GP that might be found out by a potential person that’s indicated with domestic violence. Also, within hospital pharmacy, quite often patients might unveil certain things about themselves, not necessarily domestic violence, but other emotional stressors or things, so I believe that hospital pharmacists just need to be equipped with. And you know the important networks of how to best manage that, or how to refer, or how to get support in place for these particular people.” [educator 04] “I think pharmacists are well placed and they’re embedded as part of the broader healthcare team, and we’re expected to screen and identify patients at risk of medication misadventure, and so we’re certainly placed to identify any additional risks to their mental health and wellbeing.” [educator 11]
	Recommendations for curriculum integration (10)	“Yeah, so just picking up the signs and then what are the students meant to do with those signs or what are the pharmacists meant to do with those signs.” [educator 01] “Also, you’d like to talk about reporting systems. Yeah, so long as I think it’s reporting to another authority or institution to say I suspect the following is going on.” [educator 03] “I think it’d be good to—because I know in the pharmacy degree they do interdisciplinary workshops where they meet with others, like nursing students and such. So I think having an interdisciplinary workshop around this would be good because that would show how all different healthcare professionals can support the one patient. That would enhance patient-centered care. So, I think that’s probably the main thing. And also, just going through the legalities of what the legal considerations of dealing with a domestic violence case, like what you can and can’t do.” [educator 08]
	Relevance of pharmacist’s role (11)	“So again, pharmacists have a scope of practice. And that scope of practice is definitely changing over time, so there’s elements with which we fit in with that scope of practice now, which we weren’t used to, like what’s happening at the moment…” [educator 02] “Other opportunities will be pharmacists could work with support workers or some specialised healthcare professionals in that area that deal with domestic violence where they provide conventional pharmacy care to those patients. Besides, there might be more nuanced pharmaceutical needs in terms of medications for instance patients with mental health conditions requiring unusual doses of psychotic medications, staged supply, Webster pack. This changes how pharmacists counsel people in terms of where they store or obtain medications from the pharmacy when there is someone in the family that can be abusive. There might be a niche role for mental health pharmacists to collaborate with an outreach mental health team within the hospital unit that is tailored towards domestic violence patients or patients with additional mental health needs.” [educator 05] “So, I think we’ve got a long way to go and it’s going to continue to evolve in advance, and as it should. And I think we need to stop limiting ourselves to traditional roles. I think we need to be able to adapt, and be able to integrate ourselves to the broader workforce in healthcare. Pharmacists have a wide array of skill sets and so we need to be able to showcase that, and be able to participate in all aspects of healthcare, beyond just the traditional kind of, you know, clinical or dispensing aspects or roles that we do.” [educator 11]
Barriers to integrating DV into the curriculum	Lack of confidence or experience (8)	“I think it’s more like that would be very hard for us to teach the students how to recognize domestic violence and intervene.” [educator 01] “can raise potential mental health stress to existing students who might have experienced DV before” [educator 05] “Another thing would probably be lack of confidence and expertise in the area. You would require, um, content experts to help develop new curriculum around the area because, for example, myself, I didn’t receive domestic violence training in my undergrad. So, Yeah, I would prefer getting input from an expert in that area.” [educator 08]
	Outside pharmacist’s current scope of practice (11)	“we are not really skilled to do that” [educator 01] “ I don’t think pharmacists should necessarily be trained to provide specific advice to people with domestic violence, but instead, they should be able to recognise and refer to the most appropriate person, whether it’s the police, social workers or other healthcare professionals.” [educator 02] “I don’t think that pharmacists have the capacity to add managing domestic violence to their course.” “It seems to me like a police, a legal and a social worker are more equipped, but certainly a pharmacist should be able to refer. I suppose that’s the main role, and that’s how I would see it.” [educator 06]
	Time constraints (11)	“Time consideration is for the development of it for who’s going to teach it that kind of thing.” [educator 03] “I think a big one is probably time and resource constraints. Um, because, you know, it takes time to develop a new curriculum that is of high quality and up to standard. Um, and so educators are already pretty time poor. We already, um, we already have a pretty densely packed curriculum. So I guess time and resource constraints are one big challenge.” [educator 08] “The most obvious barrier would be that the current pharmacy curriculum has a lot of stuff in there already, i.e.,: clinical and non-clinical aspect, thus trying to fit something (DV) in, might mean that we should defocus on certain areas, which also depends on how much focus we need to put on DV.” [educator 05]
Ethical or legal consideration related to a pharmacist’s role and responsibilities	Ethical responsibilities (8)	“I think from an ethical perspective, it’s important to have the appropriate skills and knowledge to recognise when domestic violence is occurring and to refer when necessary.” [educator 02] “I guess in terms of ethical considerations and legal considerations. I think, yeah, like the most important thing would be to follow, I guess, evidence-based guidelines. And yeah, making sure that particular work that’s been done around domestic violence that’s incorporated into the curriculum.” [educator 04]
	Legal considerations (9)	“From a legal perspective, if we’re not trained, we shouldn’t be providing legal assistance for a person that’s undergoing domestic violence. For example, because we’re not lawyers, we’re pharmacists. So there’s a limit to the amount of care that we provide based on our expertise and so on. I see a legal consideration in terms of overstepping the mark from a pharmacist perspective and giving advice on aspects that we are not qualified to do.” [educator 02] “I guess mostly on the legal side it should align with professional standards and guidelines. Also incorporate accurate and evidence-based learning, so using reliable resources. If you’re using any case studies, make sure that whoever is the subject of a case study is kept confidential. So, confidentiality and privacy.” [educator 08] “Other kinds of considerations from a legal or ethical perspective would be the pharmacist’s responsibility if they do identify at risk patients or patients that are under experiencing domestic violence, what are their responsibilities in terms of mandatory reporting and how they escalate that. And I guess where that’s a little bit more grey in terms of what their legal obligations are, and again, I’m bringing it back to scope of practice.” [educator 11]
	Lack of clarity on ethical and legal considerations (3)	“I think maybe as well, you could run into maybe legal issues, as I said if there are maybe some warrants or some you know they have already got restrictions or not and you are saying something in a lecture that you have kind of advocated for, for the students to do within their own household. That they are not allowed to do or they will get in trouble for.” [educator 01]
Understanding of DV related to a pharmacist’s role and responsibilities	Definitions and interpretations of DV (12)	“I guess violence in the home, so it could be verbal or physical abuse and maybe one of the household members feeling scared of the other.” [educator 01] “My understanding of domestic violence, so how I define it would be any sort of violence, whether it be physical or emotional, that occurs in a person’s own household.” [educator 02] “Um, so I guess I would perceive domestic violence as violent or abusive behaviour directed by one family member or household member or spouse, against another. So, within a household.” [08] “Domestic violence is any sort of danger or harm that an individual is being placed in their home environment. So whether that be from a spouse or family member, or someone that they live with. I guess placing at risk, whether that be physical harm or psychological harm.” [educator 10]
Pharmacist resources on DV	Familiarity with available resources (8)	“There are resources for patients who require mental health needs in general like Better Help to contact and other counselling services.” [educator 05] “I know um there’s 1-800-RESPECT. It’s like a hotline for people going through domestic violence. So I’m sure they have resources available. But, like a Google search also gave me RACGP, which is the Royal Australian College of General Practitioners, their white book, which is like a handbook for GPs on dealing with domestic violence cases.” educator [08] “There’s a lot of training providers that specialise in domestic violence training like Safe and Equal and 1-800-RESPECT.” [educator 09] “I guess this is specific to Victoria, but overall, the Department of Health releases a number of guidelines, training modules, and resources as well, not just for pharmacists, but for all healthcare professionals, particularly the ones working in mental health.” [educator 11] “I think that there are things out there, like, I think there’s government programs at the moment, like where they’ve developed resources for health professionals. So, I think it’s sort of like more resources are becoming available. And there’s a lot of stuff out there that we can use. It’s not like the pharmacy faculty has to start from scratch.” [educator 2]
	Uncertainty about available resources (4)	“I don’t really know. It’s not really my area of expertise. But I would have thought there would be information from the government on this issue. But yeah I’m not sure what resources are available, to be perfectly honest with you, I’m not familiar with any off the top of my head.” [educator 02] “I’m pretty sure there’s plenty of resources out there that can be accessed.” [educator 03]
